# Chromoanasynthesis is a common mechanism that leads to *ERBB2* amplifications in a cohort of early stage HER2^+^ breast cancer samples

**DOI:** 10.1186/s12885-018-4594-0

**Published:** 2018-07-13

**Authors:** George Vasmatzis, Xue Wang, James B. Smadbeck, Stephen J. Murphy, Katherine B. Geiersbach, Sarah H. Johnson, Athanasios G. Gaitatzes, Yan W. Asmann, Farhad Kosari, Mitesh J. Borad, Daniel J. Serie, Sarah A. McLaughlin, Jennifer M. Kachergus, Brian M. Necela, E. Aubrey Thompson

**Affiliations:** 1 Department of Molecular Medicine, Mayo Clinic, 200 First St., SE, Rochester, MN 55905 USA; 2 Center for Individualized Medicine, Mayo Clinic, 200 First St., SE, Rochester, MN 55905 USA; 3 https://www.wholegenome.io; 4 Health Sciences Research, Mayo Clinic, Jacksonville, Florida USA; 5 Laboratory Medicine and Pathology, Mayo Clinic, Rochester, MN USA; 6 Hematology/Oncology, Mayo Clinic, Phoenix, Arizona USA; 7 General Surgery, Mayo Clinic, Jacksonville, Florida USA; 8 Cancer Research, Mayo Clinic, Jacksonville, Florida USA; 9 Cancer Biology, Mayo Clinic, Griffin Building 214, Jacksonville, Florida USA

**Keywords:** Amplification, Replication, Chromothripsis, Chromoplexy, Chromoanagenesis, Chromoanasynthesis, Chromodesmy, Neochromosome

## Abstract

**Background:**

HER2 positive (HER2+) breast cancers involve chromosomal structural alterations that act as oncogenic driver events.

**Methods:**

We interrogated the genomic structure of 18 clinically-defined HER2+ breast tumors through integrated analysis of whole genome and transcriptome sequencing, coupled with clinical information.

**Results:**

*ERBB2* overexpression in 15 of these tumors was associated with *ERBB2* amplification due to chromoanasynthesis with six of them containing single events and the other nine exhibiting multiple events. Two of the more complex cases had adverse clinical outcomes. Chromosomes 8 was commonly involved in the same chromoanasynthesis with 17. In ten cases where chromosome 8 was involved we observed *NRG1* fusions (two cases), *NRG1* amplification (one case), *FGFR1* amplification and *ADAM32* or *ADAM5* fusions. *ERBB3* over-expression was associated with *NRG1* fusions and *EGFR* and *ERBB3* expressions were anti-correlated. Of the remaining three cases, one had a small duplication fully encompassing *ERBB2* and was accompanied with a pathogenic mutation.

**Conclusion:**

Chromoanasynthesis involving chromosome 17 can lead to *ERBB2* amplifications in HER2+ breast cancer. However, additional large genomic alterations contribute to a high level of genomic complexity, generating the hypothesis that worse outcome could be associated with multiple chromoanasynthetic events.

**Electronic supplementary material:**

The online version of this article (10.1186/s12885-018-4594-0) contains supplementary material, which is available to authorized users.

## Background

Large genomic rearrangements have emerged as a potential source of oncogenic driver mutations in breast cancer [1]. The classical example of the role of large genomic rearrangements as oncogenic drivers is HER2+ breast cancer in which the *ERBB2* gene (encoding the HER2 receptor subunit) is amplified along with several other genes in the vicinity of chromosome 17q12. The *ERBB2* gene is an important oncogenic driver in at least 15% of invasive breast cancers. Amplification of *ERBB2* at the DNA level leads to over expression of HER2 receptor tyrosine kinase protein on the cell surface [2], which is believed to drive malignant transformation due to hyper-activation of downstream signaling pathways that impinge upon proliferation and survival. Breast cancers with *ERBB2* gene amplification were associated with a poor prognosis prior to the availability of HER2-targeted therapy [3, 4].

The clinical relevance of the HER2 receptor increased with the development of Herceptin (trastuzumab), the first HER2-tageted monoclonal antibody therapy for treating patients with breast cancer [5, 6]. Patients treated with Herceptin showed improved survival in early clinical trials, paving the way for the clinical use of HER2-targeted therapy [7–9]. Current therapeutic options for HER2+ breast tumors target the HER2 receptor using either monocolonal antibodies (e.g. trastuzumab, pertuzumab) or small molecule receptor tyrosine kinase inhibitors (e.g. lapatinib, neratinib). Early stage HER2+ tumors are quite responsive to such therapy, with long term recurrence free survival achieved in 75–80% of patients [7–10].

The definition of what constitutes clinically significant *ERBB2* amplification or HER2 overexpression has evolved over the years since the discovery of this biomarker. For in situ hybridization methods, Slamon et al. initially used the *ERBB2* copy number (> 5 copies as amplified) [2, 3], and later the HER2/chr17 centromere ratio became the preferred determinant of HER2 amplification status, with a ratio of 2.0 or greater defining amplification [11]. Immunohistochemistry was used to identify overexpression, with evolving definitions for the minimum level of staining intensity and the staining pattern on the cell membrane. Guidelines for HER2 testing in breast cancer have been published by an expert panel with members of ASCO (American Society of Clinical Oncology) and CAP (College of American Pathologists) to standardize clinical testing and to refine the criteria for a positive HER2 result [12, 13]. These observations raise two important questions. First, from the standpoint of tumor biology, what features generally define the genomic architecture of HER2+ tumors, with particular regard for large genomic rearrangements that may extend beyond the *ERBB2* amplicon? Second, from a clinical-translational perspective, to what extent do these large chromosomal rearrangements contribute to genomic complexity that might account, at least in part, for the 20–25% recurrence rates after HER2-targeted therapy?

Gene amplifications commonly arise from replications of small regions of the genome. Possible mechanisms include the generation of either multiple tandem duplications or acentric extrachromosomal DNA circles [14]. However, more recently through the analysis of whole genome sequence data, these focal amplifications were frequently observed associated with complex chromosomal shuffling events [14]. These complex-shuffling events involved either one chromosome or two or even more chromosomes and they have been referred to as chromothripsis [15, 16] or chromoplexy [17]. Structurally, this process resembles single or multiple chromosomal knotting that eventually results in generation of one or more new chromosomes that contain genomic information from the parent chromosomes. The result of this process is often referred to as chromoanagenesis [18] or chromoanasynthesis [19, 20]. The newly formed chromosome is now susceptible to focal replication that is likely driven by selective advantage conveyed by the generation of oncogenic drivers. To reconcile the definitions of this phenomenon for the purpose of this paper we decided to use a new term “chromodesmy” to encapsulate all the different generating mechanisms and the term “chromoanasynthesis” to encapsulate the mechanism that leads to the resulting new chromosomes, which below will be referred to as “neochromosomes”.

The extent to which these processes are linked to *ERBB2* amplification in HER2+ breast cancer is largely unknown. We analyzed 18 HER2+ breast tumors using a combination of mate-pair genomic sequencing (MPseq) [21–23], RNA sequence analysis (RNAseq), and NanoString 3D Biology ™ to assess the extent to which large chromosomal alterations both within and outside of chr 17q12 are observed and are associated with *ERBB2* amplification and overexpression. The data reveal that chromodesmic processes involving chromosome 8 and chromosome 17 are commonly, but not invariably, associated with *ERBB2* amplification and overexpression.

## Methods

The aim of this study was to interrogate the genomic structure of 18 HER2+ breast tumors through integrated analysis of whole genome and transcriptome sequencing, coupled with clinical information. All tumors specimens were obtained from the Mayo Clinic biobank and the study was performed under full Mayo Clinic Institutional Review Board (IRB) approval with written consent obtained from all patients. Biospecimen handling information as outlined by the BRISQ criteria [24], is included as a supplemental table (Additional file 1: Table S1).

All tumors were clinically defined as HER2+ by ASCO/CAP guidelines: HER2 IHC 3+ and/or FISH > 2.0 in > 10% of tumor cells. HER2 IHC and *ERBB2* FISH were performed by Mayo Medical Laboratories as part of the patient’s routine breast cancer diagnosis. Fresh frozen tumor specimens were cryo-sectioned and RNA and DNA extracted using routine protocols [25]. RNA integrity was evaluated by Agilent Bioanalyzer and RINs > 8.0 were observed for all samples subjected to RNAseq analysis. An alpha version of the NanoString 3D Biology ™ platform was used to assess HER2 protein abundance.

MPseq was used to detect structural variants at gene level resolution through its specialized whole genome tiling with larger 2-5 kb fragment derived DNA libraries [26–35]. MPseq and RNAseq transcriptomic analysis were performed on 24 HER2+ breast cancer samples. Four samples had insufficient tumor (< 10%) to define rearrangements and were excluded from further analysis. Two samples were excluded due to ambiguities in the tumor registry. Indexed libraries for MPseq (1μg DNA) and RNAseq (150 ng total RNA) were generated using the Nextera Mate-Pair Kit (Illumina, CA, FC-132-1001) or the TruSeq RNA Library Prep Kit v2 (Illumina, CA, RS-122-2001), following the manufacturer’s instructions. Libraries were sequenced on the Illumina HiSeq2000 platform at a depth of three or six libraries per lane, respectively.

### Detection of structural variants

BIMA, developed by Biomarker Discovery Lab at Mayo Clinic, mapped all MPseq fragments to GRCh38. BIMA is a binary indexing algorithm for simultaneous mapping of both reads in a fragment, specially designed for MPseq [26]. Structural variants were detected using SVAtools, version 0.34.16,, a suite of algorithms also developed by the Biomarker Discovery Lab at Mayo Clinic [22, 23]. Four main components of SVAtools includes: 1) junction detection with a customized rapid clustering algorithm to detect discordant fragments supporting a common junction, 2) a system of masks and filters to remove false-positive junctions. The mask primarily eliminates normal structural variants not present in the reference genome and eliminates mapping artifacts due to repeat or un-sequenced genomic regions. The filters use BIMA mapping scores to identify NGS library prep artifacts and to eliminate poorly qualified breakpoints. 3) CNV detection, using the read count of concordant fragments within non-overlapping bins. The detected junctions provide enhanced edge detection resolution and sensitivity. 4) Visualization of all structural variants via genome plots [21]. Putative junctions as well as any two genomic regions of interest can be visualized and further inspected via junction plots and region plots, illustrations of all fragments mapping within and between two genomic regions.

Following junction detection SVAtools’ CNV detection was used to determine the location and depth of copy number variation across the genome. This algorithm uses both a sliding window statistical method to determine likely copy number edges from read depth, as well as using breakpoints locations determined in the junction detection stage to more accurately place these edges. Once the genome was segmented into likely copy number regions, the normalized read depth (NRD) was calculated as the read depth within the region was divided by the expected read depth for normal diploid level for the sample. Regions with NRD scores that deviated significantly from the expected diploid level (NRD = 2.0) were called a copy number variant. This NRD score was used to estimate the level of amplification in a region according to the following equation:

$$ {X}_i=\frac{\left|{NRD}_i-{NRD}^{\prime}\right|}{\tau } $$

Xi is the change in copy number for a region, NRDi is the NRD value calculated for the region, NRD’ is the expected normal diploid NRD level, and τ is the fraction of tumor cells in the sample. Tumor fraction was determined by calculating the cumulative NRD score for all regions called a loss in a sample. The difference between this cumulative NRD score and expected diploid level (NRD = 2.0) was the tumor fraction. If the deleted regions were not enough to calculate the tumor fraction the gained CNV regions were used. If neither was available then the tumor fraction was considered indeterminate.

A tumor was denoted as chromoanasynthetic if 5 or more junctions were found between any two chromosomes.

#### RNAseq

RNA-sequencing libraries for 18 samples were prepared according to the Illumina truseq protocol and run on the HiSeq2000 platform. The RNA-Seq Paired end sequence data were reran using MAP-RSeq version 3.0.0 [36], an integrated RNA-Seq bioinformatics pipeline developed at the Mayo Clinic for comprehensive analysis of raw RNA sequencing paired-end reads. MAP-RSeq employs the very fast, accurate and splice-aware aligner, STAR [37], to align reads to the reference human genome, build hg38. The aligned reads are then processed through a variety of modules in a parallel fashion. Gene and exon expression quantification is performed using the Subread [38] package to obtain both raw and normalized (RPKM – Reads Per Kilobase per Million mapped) reads. Finally, the “.count” files from the previous step were used by the edgeR (version 3.16.5, R 3.3.1) program to generate a normalized expression matrix for all samples. The *ERBB2* mRNA abundance was extracted from the normalized RNAseq data.

#### NanoString 3D biology™

An alpha version of the 3D Biology platform was used to assess mRNA abundance (Pan Cancer Pathways), protein expression (including *ERBB2*), and single nucleotide variants. Data were analyzed using the nSolver Advanced Applications software.

## Results

MPseq is a very efficient method to examine the rearrangement landscape of tumor cells. We focused in chromosomal junctions (connections between distant breakpoint of the genome), to investigate how *ERBB2* amplifications arise in HER2+ breast cancer. All the examined tumors exhibited aberrant junctions. The total junction counts of all discordantly mapping genomic breakpoints detected in the 18 clinically determined HER2+ cases successfully analyzed by MPseq varied from 31 to 400 (Table 1). Out of these samples, 16 exhibited extensive chromoanasynthesis (Table 1).

**Table 1:**
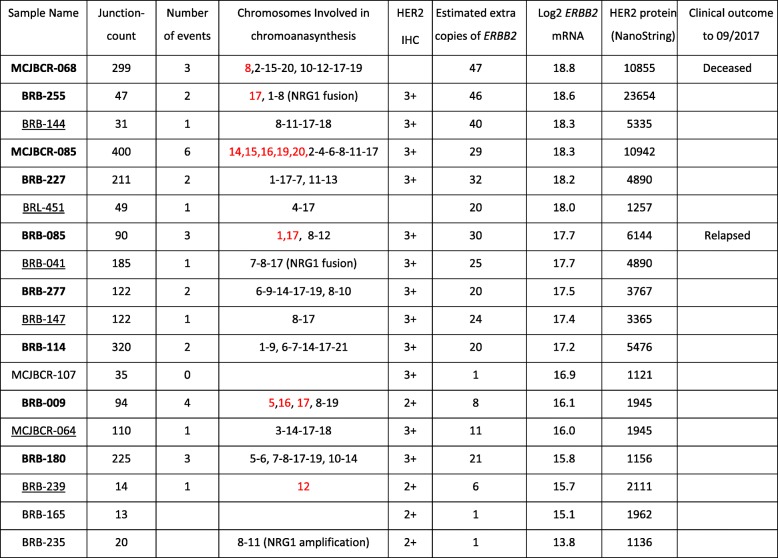
Summary of molecular data in all cases analyzed. The Junction-count field displays the number of junctions with 4 or more associates. The number of events field displays the number of independent chomoanasynthetic events. The chromosome number when the chromoanasynthesis is confined in that chromosome is color-coded red

**Fig. 1:**
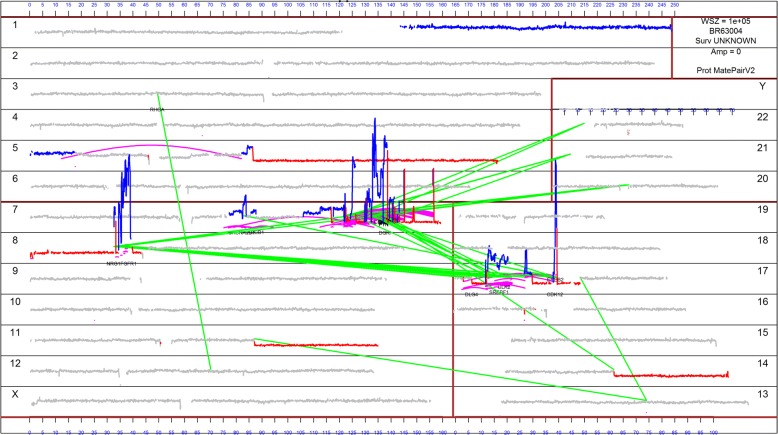
Genome U-plot representation of case BRB-041. Coverage of each chromosome is presented horizontally with grey regions indicating normal diploid coverage. Red and blue chromosomal regions indicate losses and gains, respectively. Straight green lines represent translocation junctions between different chromosomes whereas magenta arcs represent junctions within chromosomes

We used a whole genome visualization layout featuring the Gnome U plot [21] to inspect the rearrangement architecture of all samples. Fig. 1 illustrates the genome plot of a representative HER2+ breast tumor (BRB-041). This tumor harbored a single 3-way chromodesmy event involving primarily chromosomes 7, 8, and 17. After this 3-way event, the tumor genome would resolve to newly synthesized neochromosomes, quite distinct from the patient’s normal diploid genome. The newly synthesized neochromosomes were deprived of large portions of 7q, 8p and 17p but showed gains and focal amplifications on the remaining portions. As a consequence of this chromodesmy, *ERBB2* was greatly amplified resulting in at least 25 additional copies. *ERBB2* was the ninth most abundant transcript by RNAseq (17.7 at the log2 scale of RPKM) in this case. The dependence of this tumor on the HER2 signaling pathway was also supported by the observed high protein expression from IHC and the Nanostring assay (Table 1).

We also examined all the other genes that could be influenced by this chromodesmy event. This event also yielded a *WIPF2-NRG1* fusion on chromosome 8, corroborated by RNAseq that lead to marked up-regulation of *NRG1* (highest *NRG1* expression among all cases). Similarly, BRAF was also amplified on chromosome 7 and lead to one of the highest BRAF expressions among all cases. Multiple other genes were influenced on chromosomes 7, 8 and 17 including an RAI1-UNC5D fusion. The rest of the tumor genome was relatively diploid and unaffected by the chromodesmy event with the exception of 1q gain, and deletions on the latter parts of the q arms of chromosomes 5, 11, and 14. The main questions that arise after examining the architecture of the case above are, first; how often chromodesmy events exist in HER2+ breast cancer, second; how consistent are these events, and third; what happens when there is no evidence of focal *ERBB2* amplification.

### Cases with *ERBB2* amplifications

**Fig. 2:**
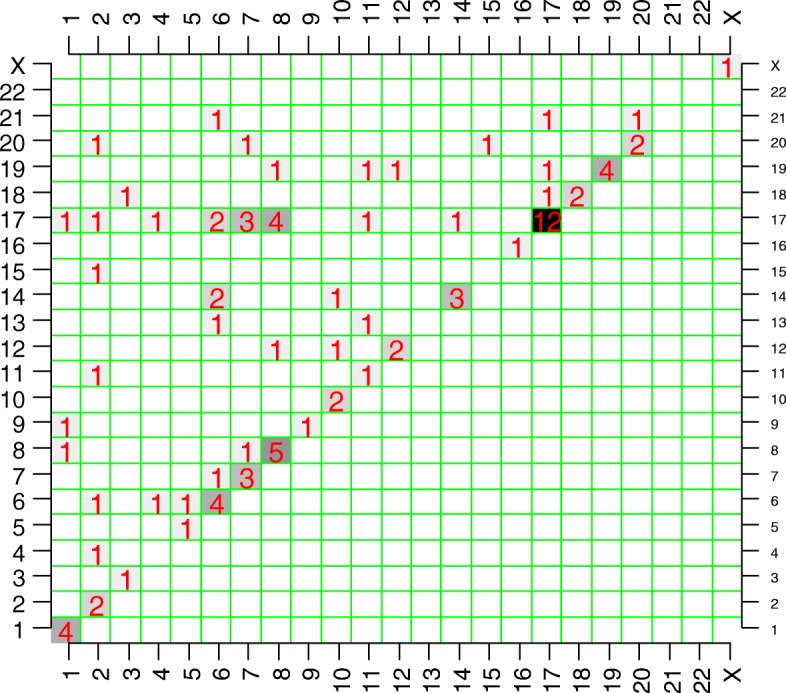
A visual synthesis of all complex junctions from all cases. The chromoanasynthetic events within and between chromosomes detected by MPseq in the 18 Breast cancer specimens are summarized above, with the numbers showing how many events involved the respective chromosomes

We then investigated the commonality of chromodesmy events in all the available samples (Additional file 1: Figure S1-S17). The most common phenomenon observed among all cases involved chromodesmy of two to six chromosomal bundles. The data are summarized in Table 1. A graphic representation of the frequency of the involved chromosomes is shown in Fig. 2. Twelve out of the 18 cases exhibit chromodesmy of multiple chromosomes that resulted in *ERBB2* amplifications. The subsequent chromoanasynthesis not only resulted in focal amplifications of *ERBB2* but also involved amplifications of other areas of the genome in all cases primarily with chromosomes 8 and 7 (Fig. 2), but less frequently with other chromosomes (see below). Two cases, BRB-255 and BRB-085, exhibit *ERBB2* amplification through intra-17 chromodesmy without an apparent involvement of other chromosomes (Additional file 1: Figure S1-S17). Finally, BRB-239 was the only case that had a classic focal *ERBB2* amplification without evidence of chromodesmy. The total number of genomically-confirmed *ERBB2*-amplified HER2+ tumors was 15. As expected, the *ERBB2*-amplified cases had the highest *ERBB2* expression (Table 1). The HER2 copy number correlated very well with the mRNA abundance (Spearman’s rho = 0.849, *p* < 0.0001) and protein expression (Spearman’s rho = 0.777, *p* = 0.0002). The only case that did not have *ERBB2* amplification but ranked 12th on the expression order was MCJBCR-107 (see below).

Additional observational analysis between cases was performed with the intention of finding commonalities and differences. The 15 *ERBB2*-amplified cases can be roughly divided into two categories; simple and complex, according to the number of coordinated chromodesmic events. The simple chromodesmy cases (underlined sample names in Table 1), like BRB-041, contained a single coordinated event that leads to amplification of *ERBB2*. The complex cases (bolded sample names in Table 1), like MCJBCR-068, corresponded to cases with multiple, potentially independent, chromodesmic events. MCJBCR-068 was one of the most complex cases with the highest *ERBB2* expression, containing three chromodesmic events and originated from a patient who relapsed and died after HER2-targeted therapy. A second patient, BRB-085, with complex events also relapsed.

Chromosome 8 rearrangements were the most frequently observed events (Table 1). Ten of the 18 cases involved events in a 30–40 Mb region of chromosome 8, associated with a number of genes that could be related to HER2 signaling, including *NRG1, FGFR1, UNC5D* and *ADAM5*. Two of these cases (BRB-041 and BRB-255) had *NRG1* fusions supported by both MPseq and RNAseq. *ERBB3* expression was also high in these two cases compared to other cases, whereas EGFR was low indicating HER3 as a likely partner of HER2 in these two cases (Additional file 1: Figure S18). Overall, *EGFR* and *ERBB3* expression were anti-correlated in the *ERBB2* amplified cases (Additional file 1: Figure S18) by a correlation coefficient − 0.56 (*p*-value = 0.025, 95% confidence interval: − 0.860 -0.085).

### Cases that did not exhibit *ERBB2* amplifications

Although, all the cases examined were clinically rendered HER2+ by IHC, they did not all show a clear amplification signal by MPseq. The remaining three HER2+ cases did not display *ERBB2* amplification at the chromosomal level. The tumor percentage in these tumor tissues ranged from 23 to 56%, which would be adequate to demonstrate amplifications. With the exception of MCJBCR-107, which ranked 12th overall in *ERBB2* mRNA abundance, the *ERBB2* expression of the remaining cases was lower than the *ERBB2*-amplified cases described above (Table 1).

A more thorough analysis of these cases revealed additional chromosomal abnormalities involving *ERBB2*. MCJBCR-107 interestingly, had a small duplication that included the *ERBB2* gene and exhibited a pathogenic variant at position chr17:39724008 (https://www.ncbi.nlm.nih.gov/SNP/snp_ref.cgi?rs=121913468, http://cancerdiscovery.aacrjournals.org/content/3/2/224.long). There are 740 reads supporting the wild type G in that case, with 2103 reads supporting the mutated T. So 75% mutated vs. 25% wild type. The mutation G- > T results in a change from Asp (D) - > Tyr (Y). The HER2 IHC clinical staining score was 3 but the FISH ratio for this tumor was 1.04. The high *ERBB2* RNA expression corroborated with strong IHC HER2 staining, the small duplication around *ERBB2*, and the pathogenic mutation points to an additional mechanism and possible biomarker for HER2+ breast cancer.

Case BRB-235 contained a gain in a large region of chromosome 17 that included *ERBB2*. It also displayed a complex event between chromosomes 8 and 11 that lead to an amplification of *NRG1*. BRB-165 gained 17q and exhibits extensive aneuploidy but no chromoanasynthesis (Additional file 1: Figure S16).

## Discussion

Early experiments using karyotyping and metaphase FISH in *ERBB2*-amplified HER2+ breast cancers showed that the classical pattern was high copy gain with the extra *ERBB2* copies residing on one or more chromosomes, typically not the chromosome of origin (chromosome 17) but another chromosome, in “homogeneously staining regions” that contained co-amplified sequences from several other chromosomes [39]. By interphase FISH analysis, the *ERBB2* signals were present as one or more clusters, rather than diffusely scattered throughout the cell, consistent with the intrachromosomal location of the amplicons [40]. Technical limitations of earlier technologies such as comparative genomic hybridization, karyotyping, and Southern blot made it impossible to determine the composition and genomic architecture of amplicons at the sequence level. More recent large scale genomic studies have further defined the spectrum of genomic abnormalities in *ERBB2*-amplified HER2+ breast cancers, but the methods used for these studies precluded a detailed analysis of the large-scale genomic architecture of *ERBB2*-containing amplicons [41, 42]. In the current study, for the first time, we have performed a detailed analysis of *ERBB2* amplification using mate pair whole genome sequencing.

Chromosomal rearrangements leading to fusions and amplifications are often the main structural drivers that lead to cancer progression and point to the targeted treatments that could benefit the patient. Amplifications such as *ERBB2* in HER2+ breast cancer also arise from structural rearrangements and can be examined using comprehensive sequencing technologies such as MPseq and RNAseq. To this end, MPseq provides a high resolution picture of the DNA structure and RNAseq can provide functional insight of how genes are expressed in the context of rearrangements.

An obvious question arises with respect to the clinically HER2+ tumors that do not appear to evidence *ERBB2* amplification or HER2 overexpression at the level of the bulk tumor. One obvious possibility might be a relatively low percentage of tumor cells in the sample. However, all of these samples exhibit gross chromosomal rearrangements, indicative of a high level of tumor cell enrichment. A more likely possibility is that these tumors are heterogeneous and comprised of a small percentage of HER2+ cells within a larger population of HER2-negative tumor cells. Recall that ASCO/CAP guidelines require that only 10% of tumor cells must be IHC 3+ for HER2 in order for the tumor to be called HER2+. However, in a bulk analysis of the sort described in this report, the relatively less abundant genomic contribution of the small percentage of HER2+ tumor cells might be obscured by the contribution from the more abundant HER2-negative tumor cells. This possibility obviously raises an interesting question about the efficacy of HER2-targeted therapy in such heterogeneous tumors. However, that question is beyond the scope of this study. Unfortunately, we do not have sufficient samples or long term follow up data to rigorously assess therapeutic outcome as a function of any of the genomic features that we have defined.

## Conclusion

Integrated DNA and RNA genomic analysis of HER2+ breast cancers, for the majority of cases, reveals that *ERBB2* amplifications are presented in the context of chromoanasynthesis involving either chromosome 17 alone, or with other chromosomes. We carefully inspected 18 clinically determined HER2+ cases with whole genome mate pair sequencing and found that 15 exhibit clear focal *ERBB2* amplification. Of those, only three cases showed amplification on 17 that did not involve any of the other chromosomes. Of the remaining 12 cases, three exhibit 2-way chromodesmy, two 3-way, four 4-way, two 5-way and one 6-way. It is unclear if all the junctions contained in these highly complex events are a result of single or multiple progressive chromosomal catastrophe events. It is much more plausible that an initial much simpler chromosomal event renders the area sensitive for subsequent junction generating events that result in gene amplifications that give advantage to cell survival and proliferation. Three other HER2+ cases by IHC displayed single copy gains on the region that included *ERBB2*, either by small duplication (in one case) or larger regions. The one sample that had a small duplication also had a pathogenic mutation.

We also observed a preferential choice of the other chromosomes involved; specifically chromosome 8 is often one of the other partners. A possible explanation is that there exist genes in areas of chromosome 8 that collaborate with *ERBB2* in the evolutionary advantage of the cells. One of these candidates is *NRG1*, a ligand of HER3, which appears to be involved in fusions associated with high expression of *NRG1* and the HER3 gene *ERBB3*. *EGFR* and *ERBB3* expressions were anti-correlated pointing towards specificity of HER2 heterodimerization with either HER1 or HER3.

## Additional file

Additional file 1: **Figure** **S1-S17.** Genome U plots of all the additional cases. Figure S18 Heat map of the *EGFR* and *ERBB3* expression log2 expression by RNAseq. Dark red indicates low expression where yellow indicates high expression. Table S1 BRISQ summary of tumor specimens. (PPTX 3536 kb)

## Abbreviations

ASCO: American Society of Clinical Oncology
CAP: College of American Pathologists
IRB: Institutional Review Board
MPseq: Mate-Pair genomic sequencing
NRD: Normalized Read Depth
RNAseq: RNA sequence analysis
RPKM: Reads Per Kilobase per Million mapped
