# Supplementary material for: Chromoanasynthesis is a common mechanism that leads to ERBB2 amplifications in a cohort of early stage HER2+ breast cancer samples
Source: BMC Cancer. 2018 Jul 13;18:738. doi: 10.1186/s12885-018-4594-0 (PMC6045826; doi:10.1186/s12885-018-4594-0)
Supplement: Supplementary file 1 — Figure S1-S17. Genome U plots of all the additional cases. Figure S18 Heat map of the EGFR and ERBB3 expression log2 expression by RNAseq. Dark red indicates low expression where yellow indicates high expression. Table S1 BRISQ summary of tumor specimens. (PPTX 3536 kb) [file 12885_2018_4594_MOESM1_ESM.pptx]

## Slide 1
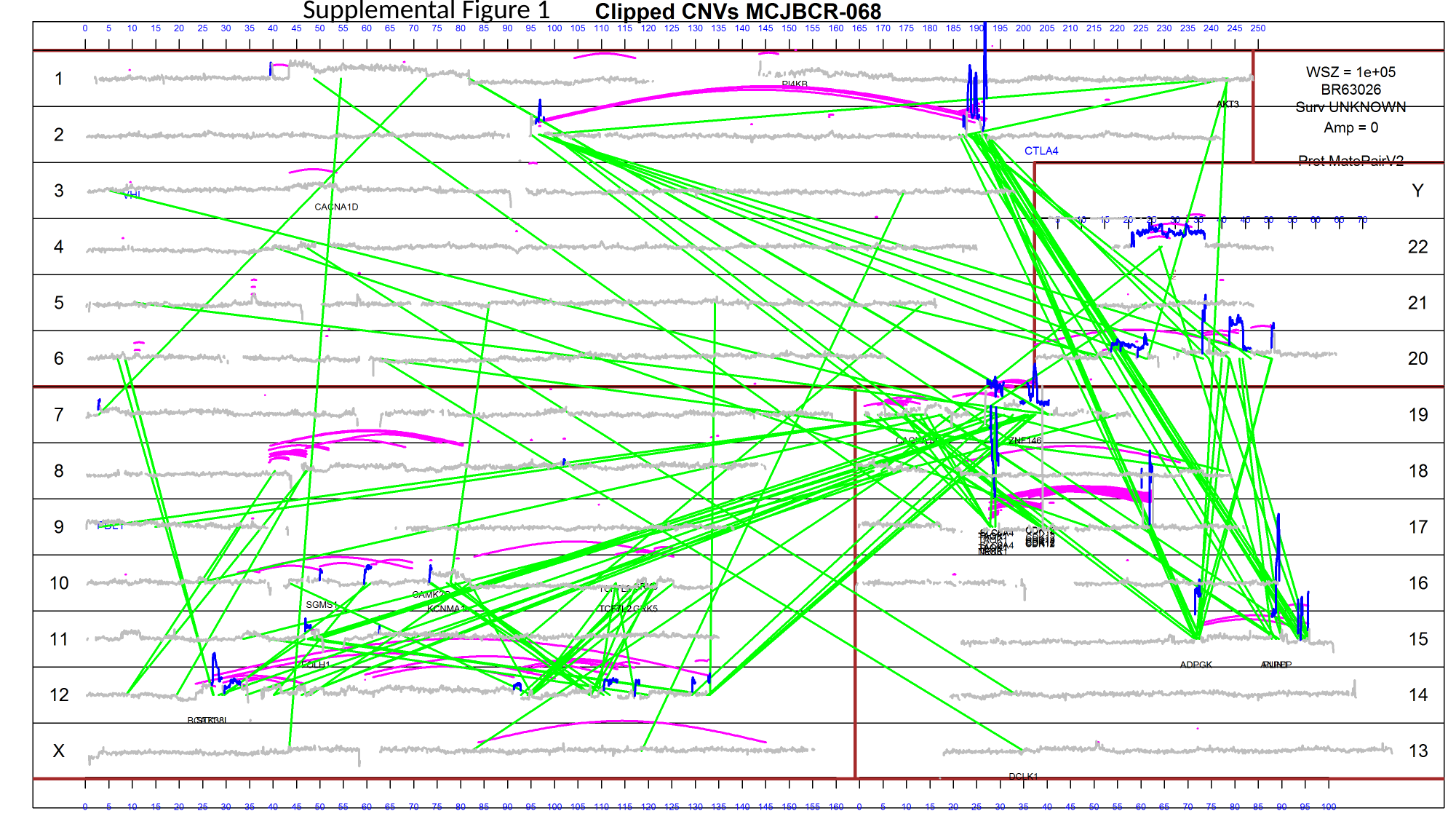

Supplemental Figure 1

## Slide 2
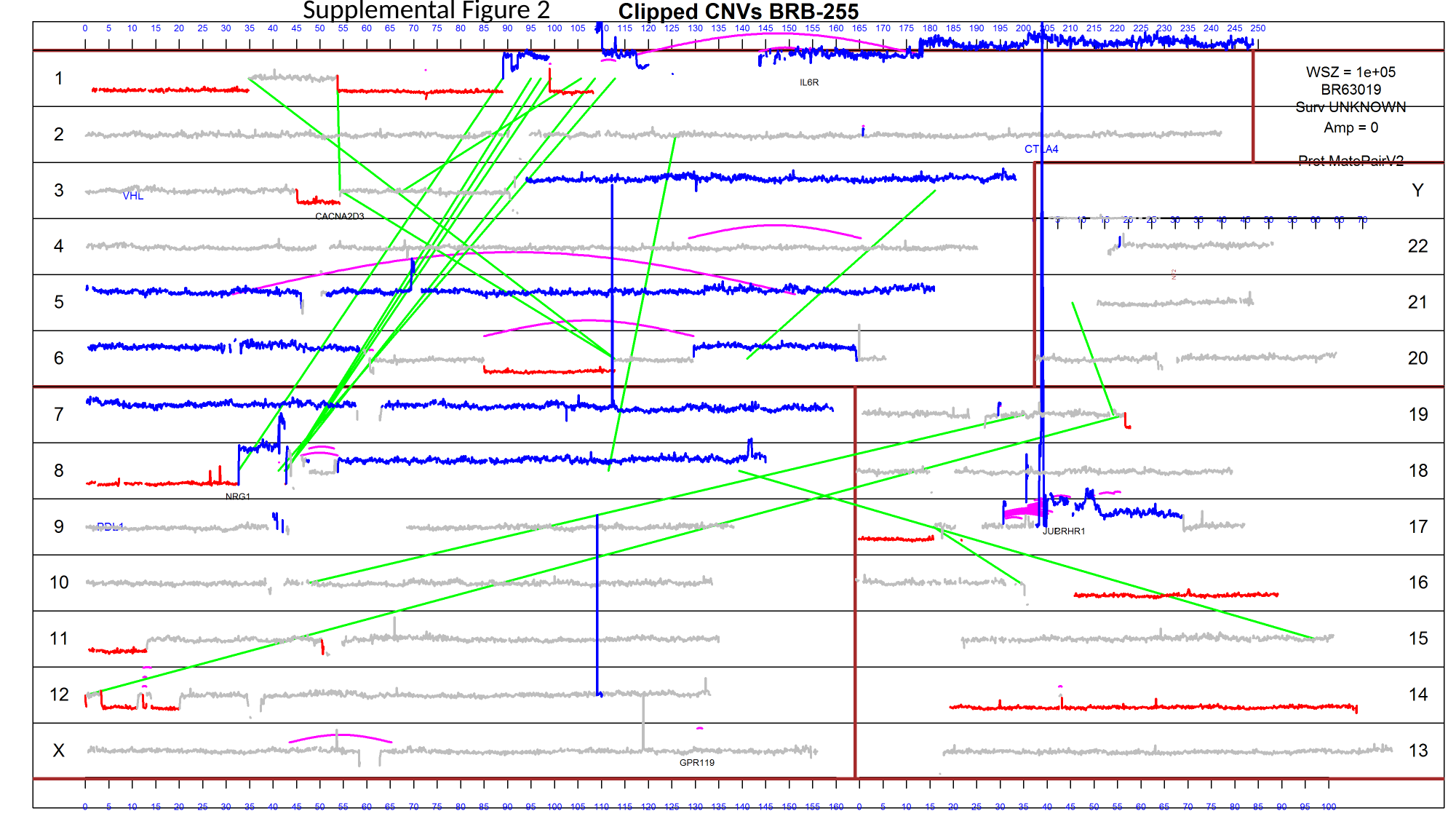

Supplemental Figure 2

## Slide 3
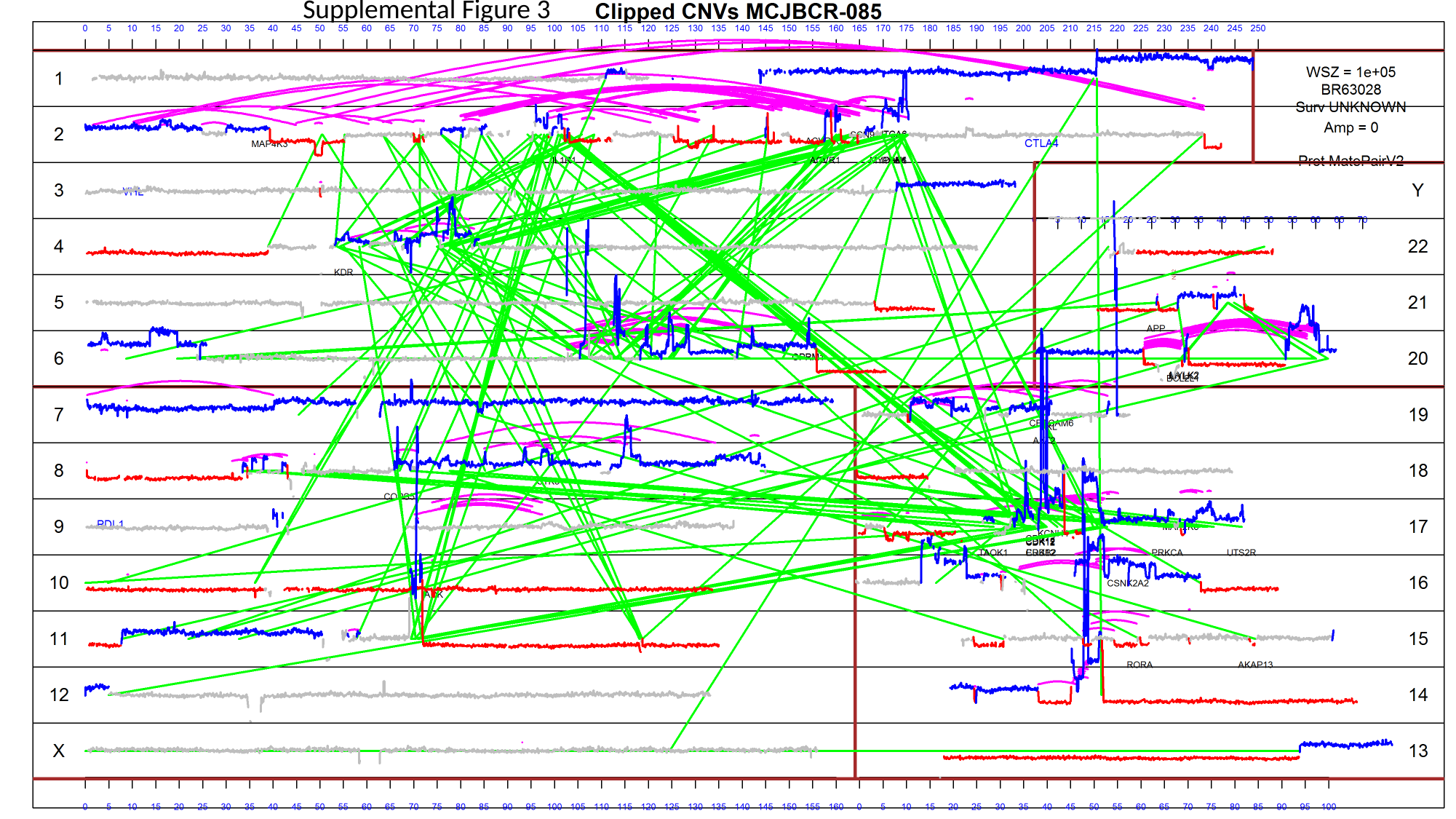

Supplemental Figure 3

## Slide 4
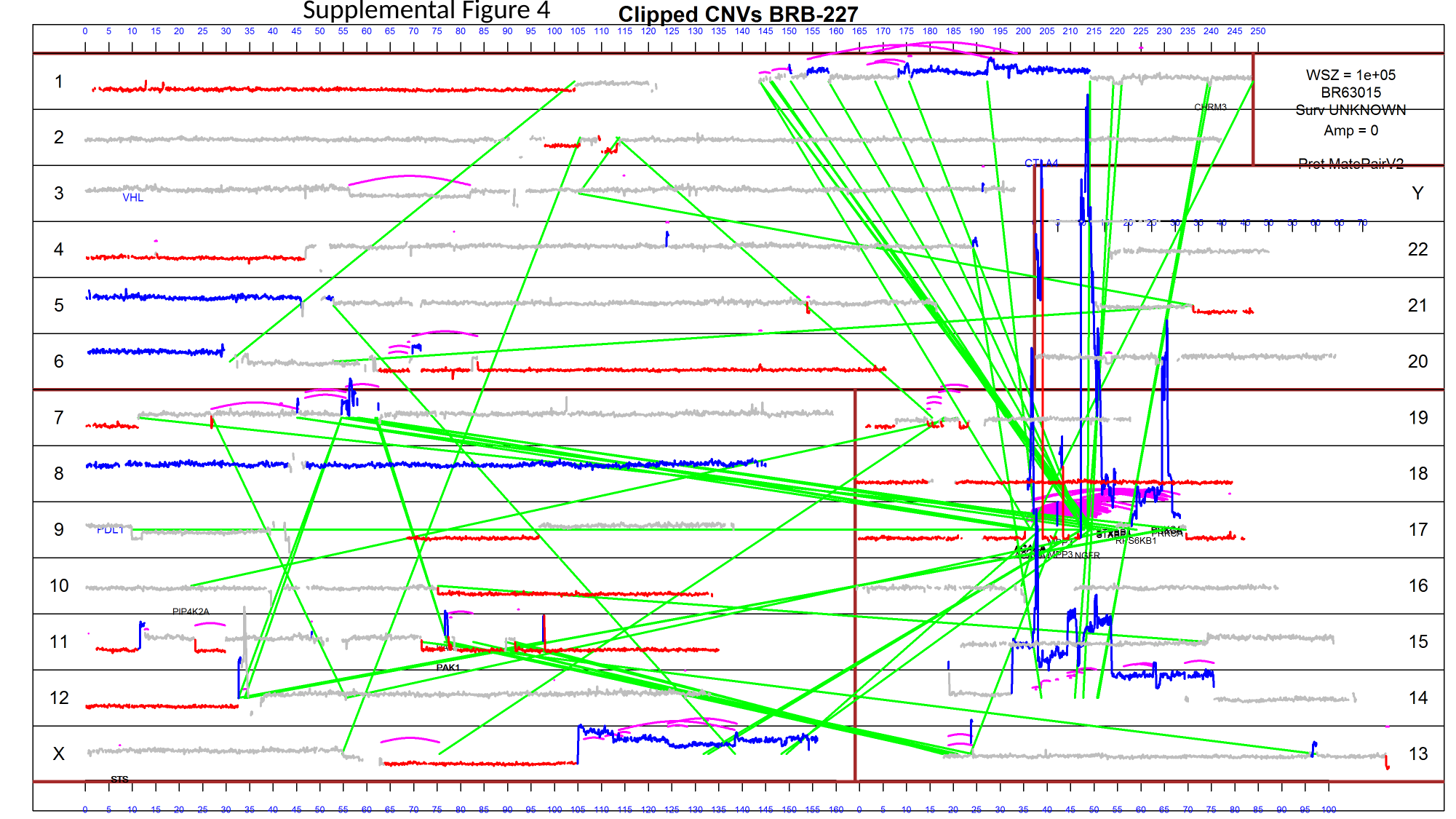

Supplemental Figure 4

## Slide 5
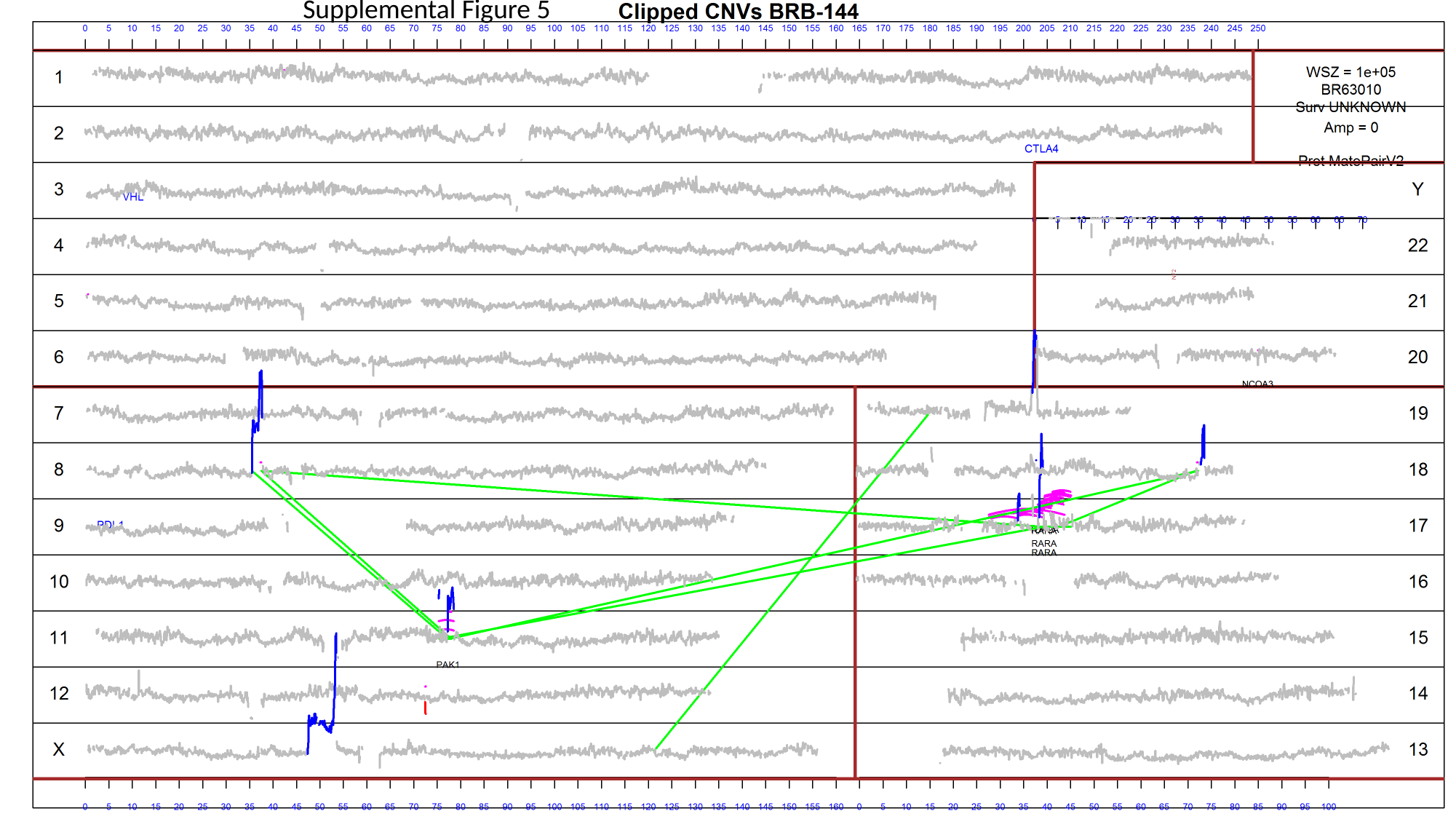

Supplemental Figure 5

## Slide 6
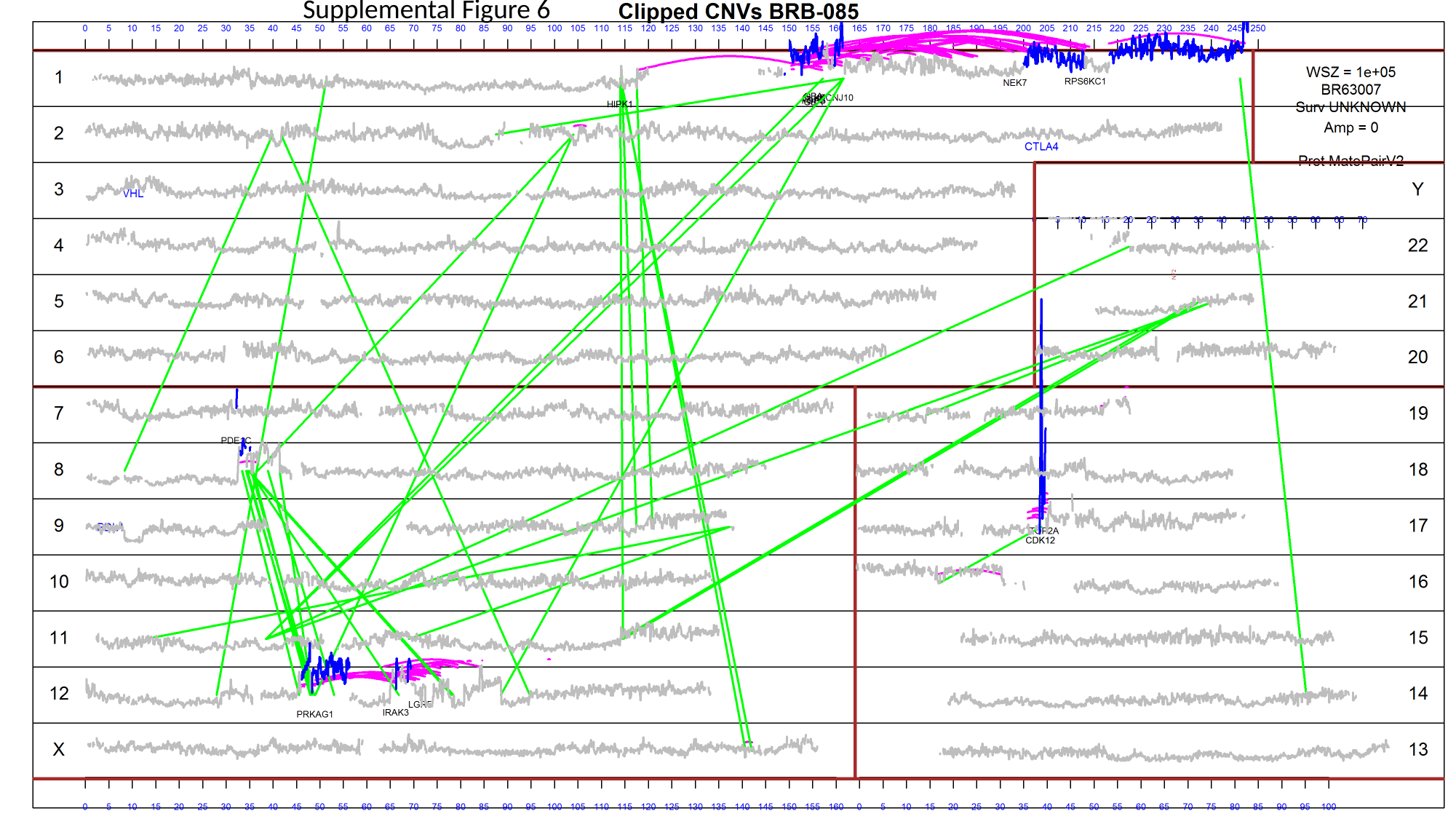

Supplemental Figure 6

## Slide 7
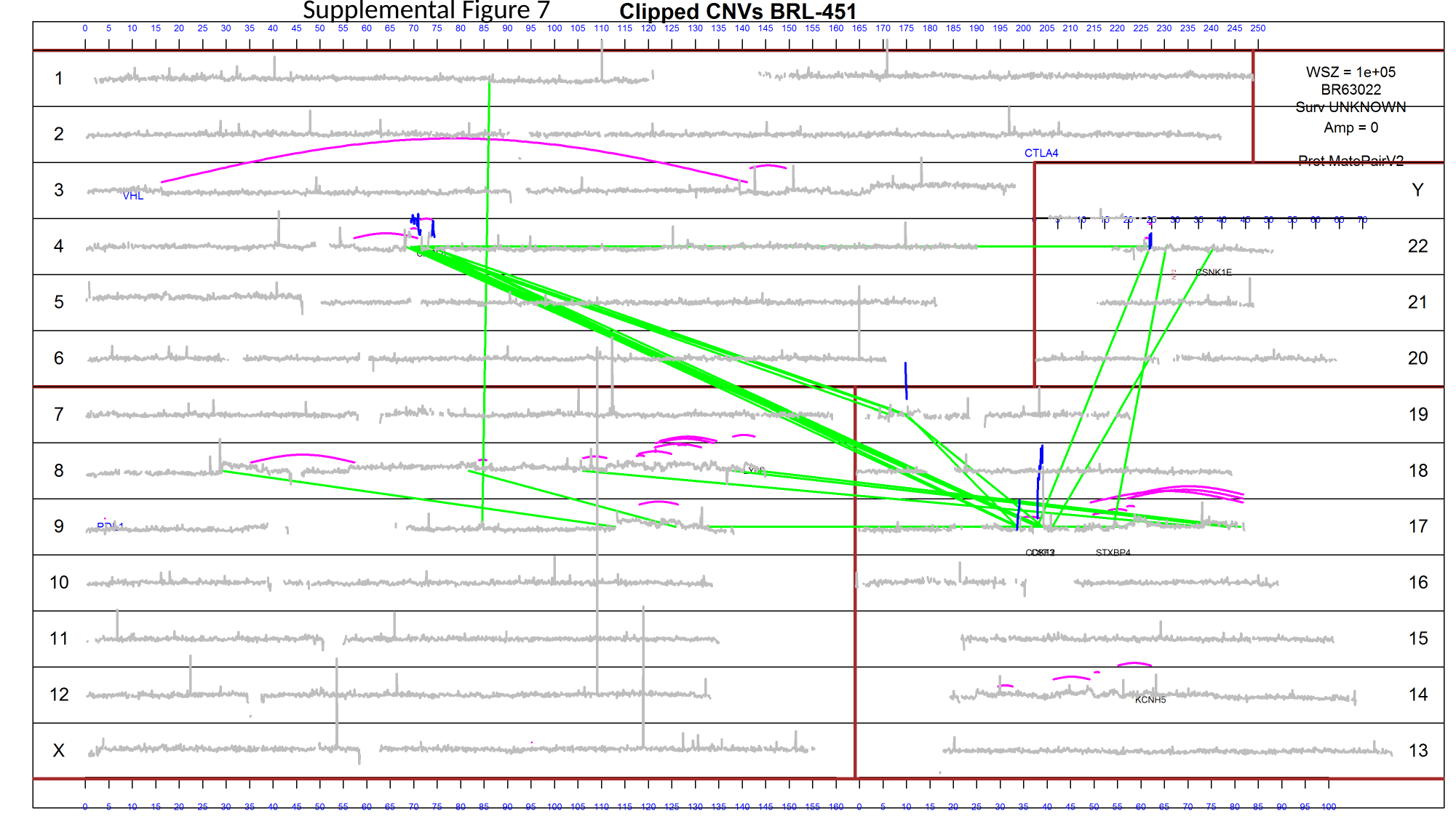

Supplemental Figure 7

## Slide 8
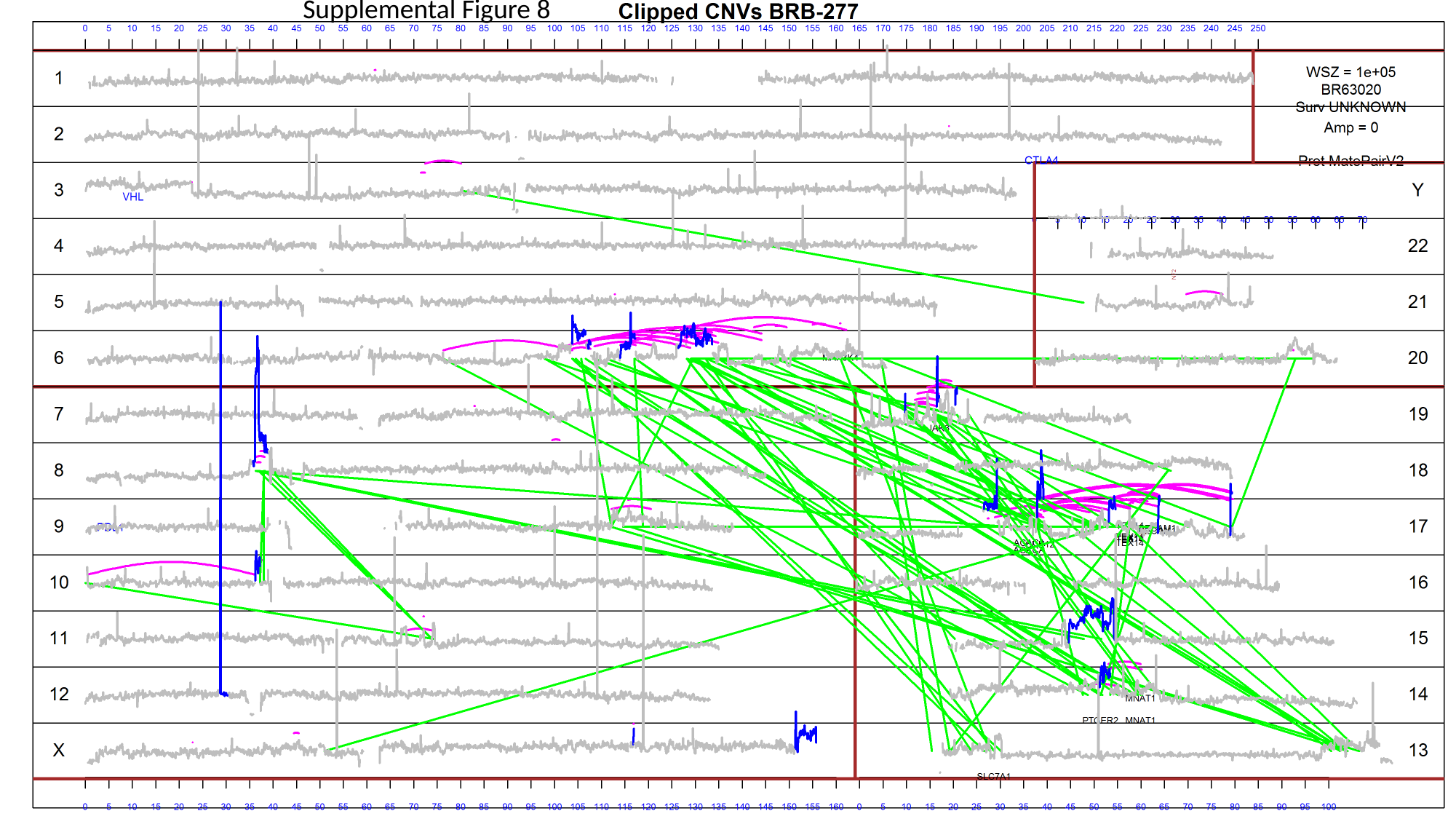

Supplemental Figure 8

## Slide 9
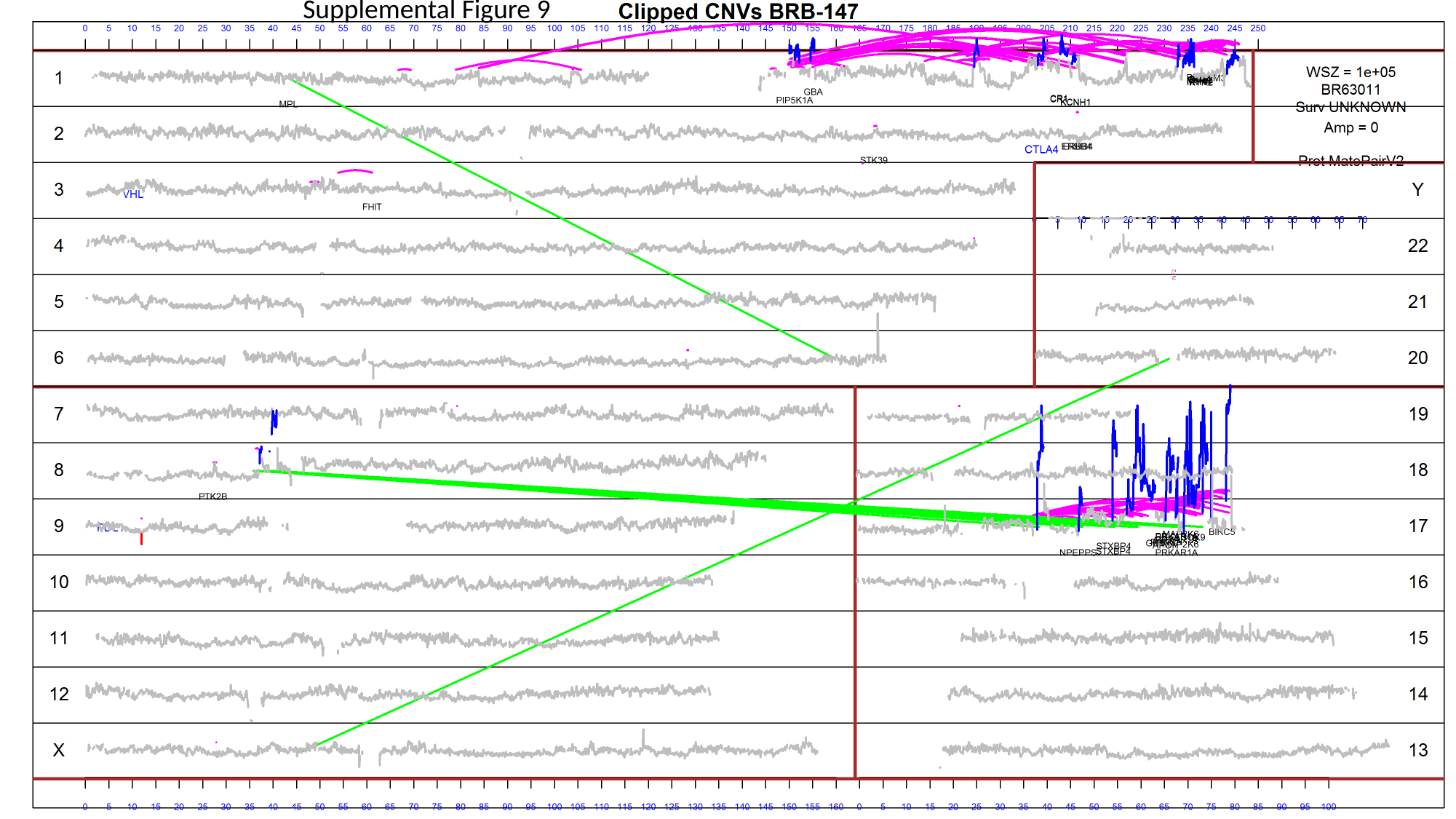

Supplemental Figure 9

## Slide 10
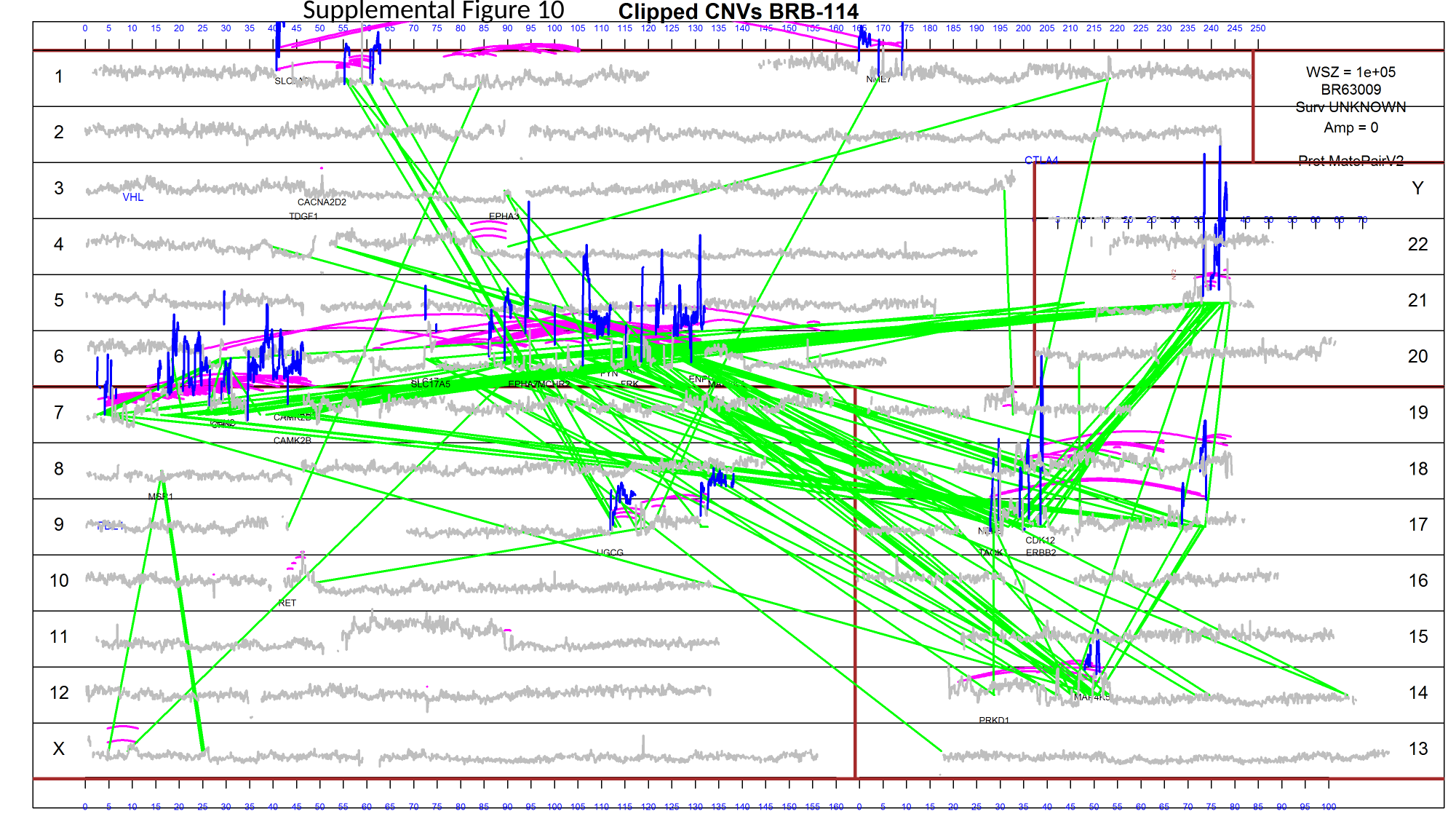

Supplemental Figure 10

## Slide 11
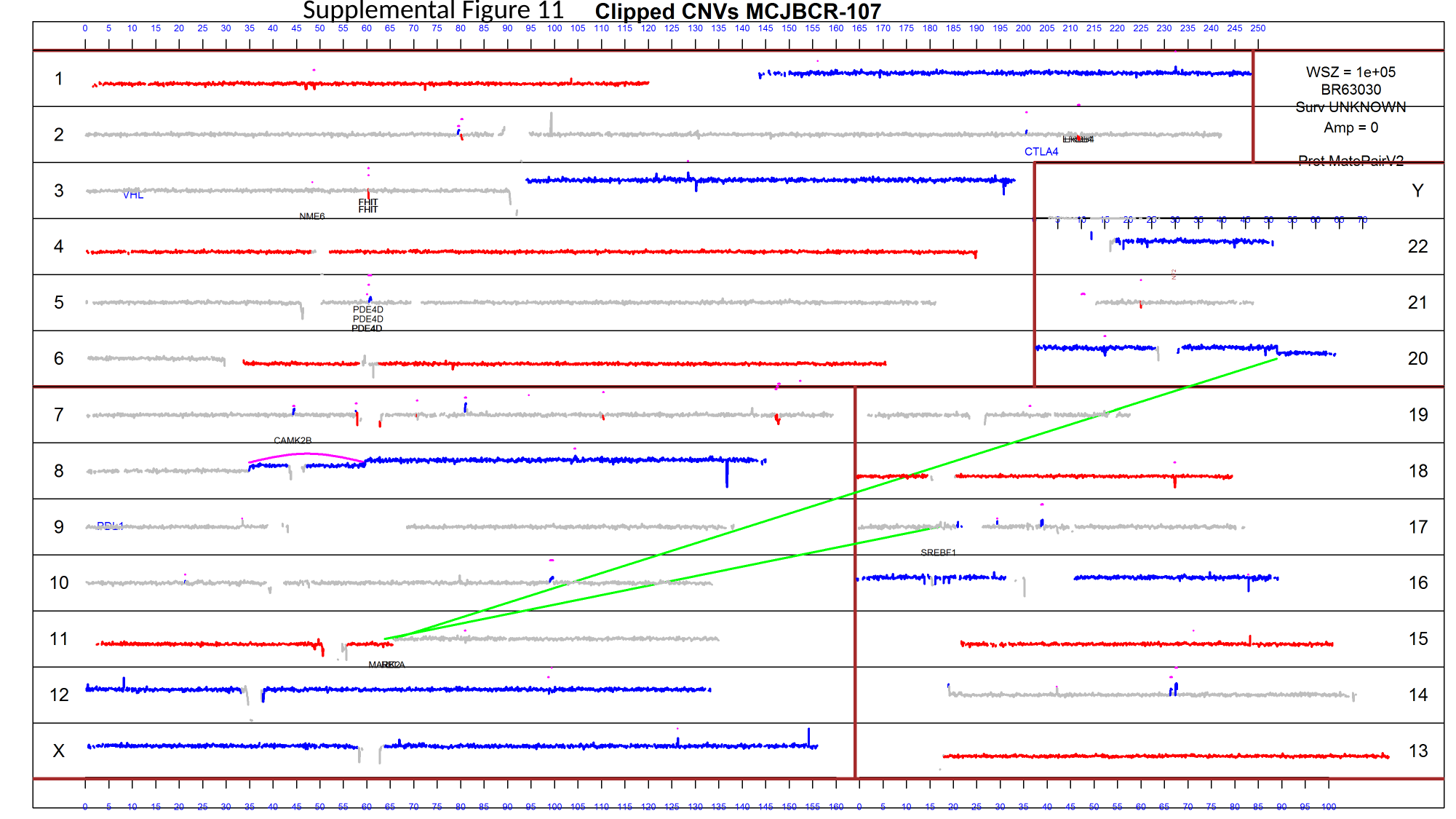

Supplemental Figure 11

## Slide 12
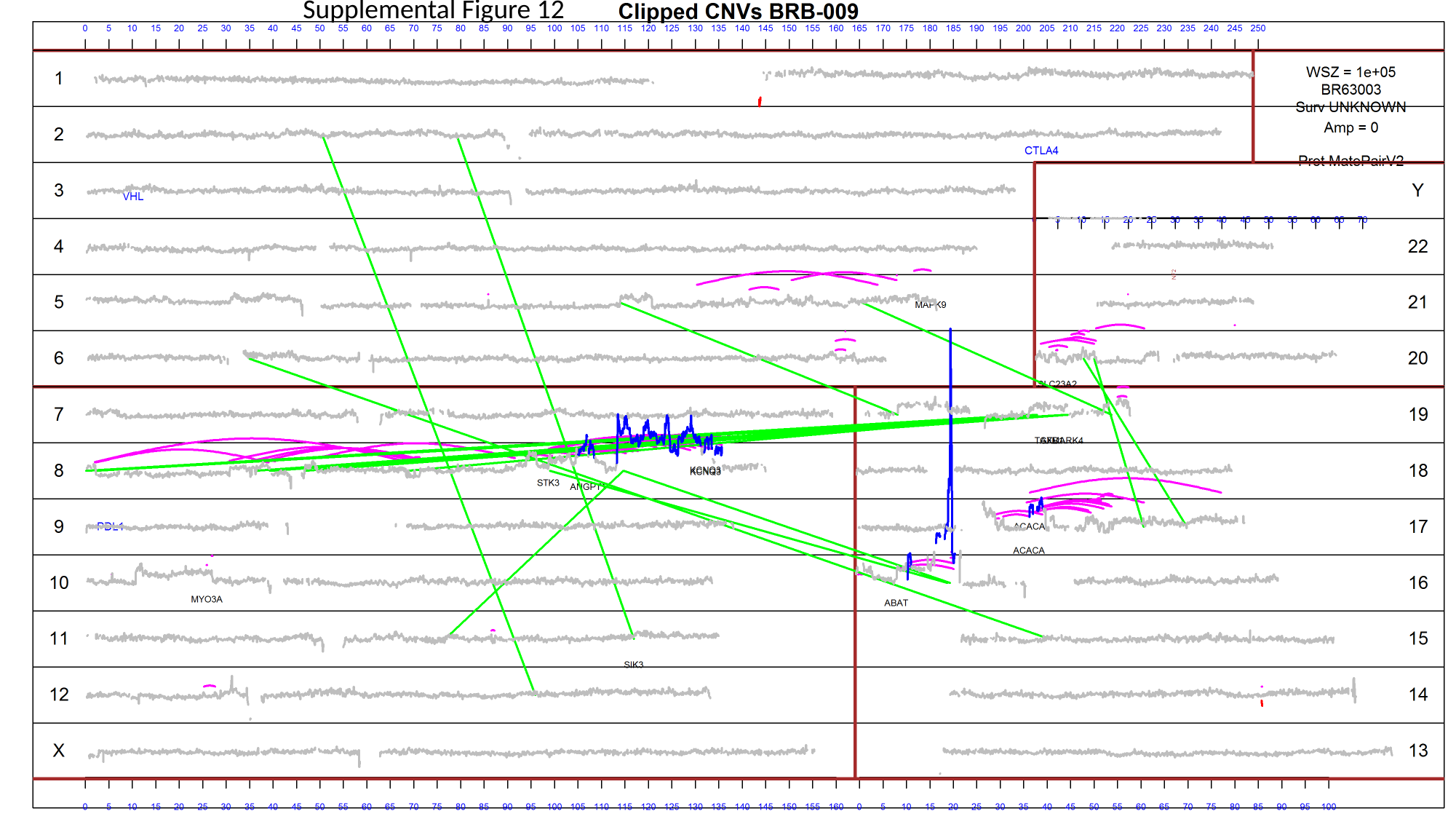

Supplemental Figure 12

## Slide 13
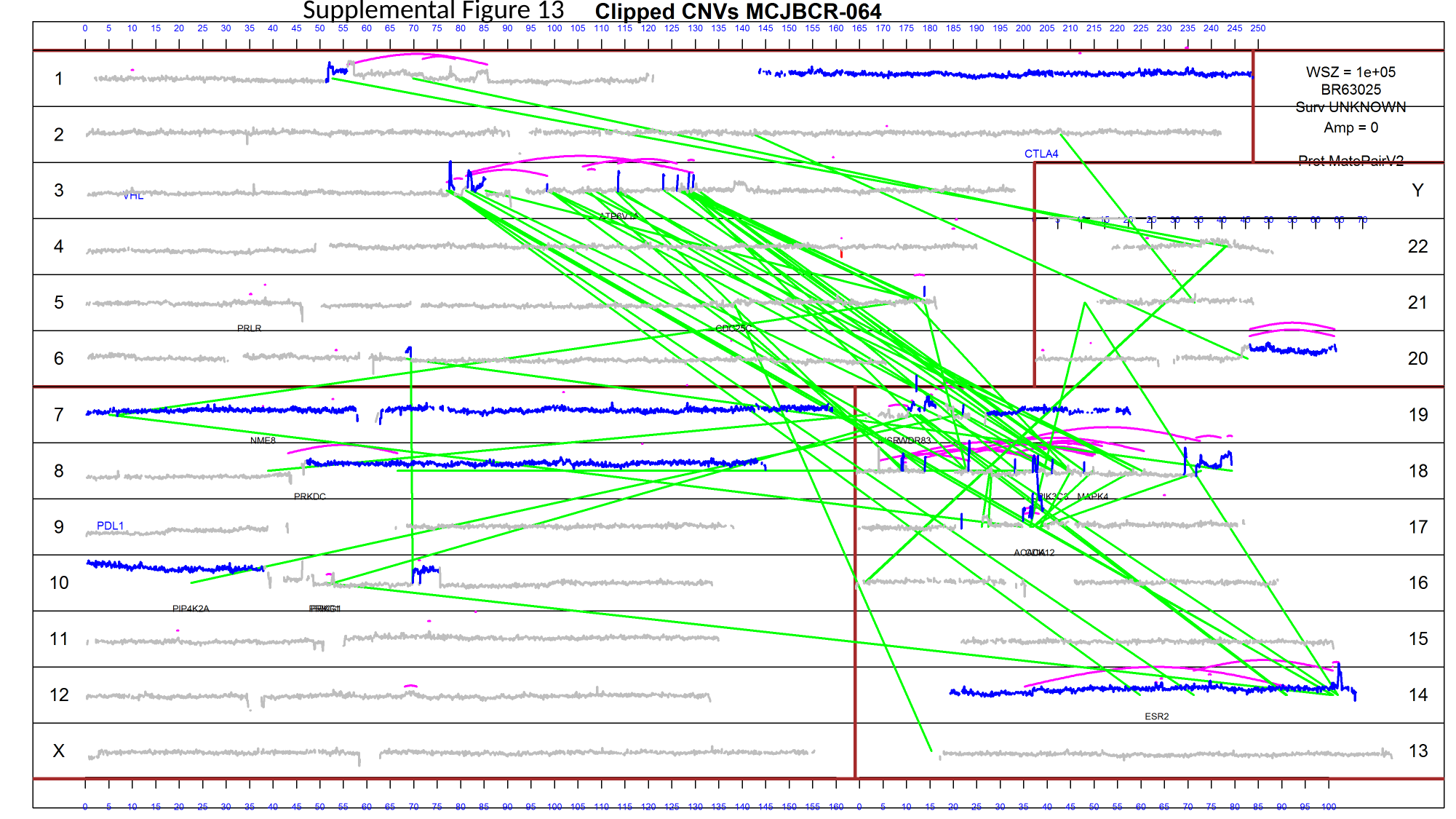

Supplemental Figure 13

## Slide 14
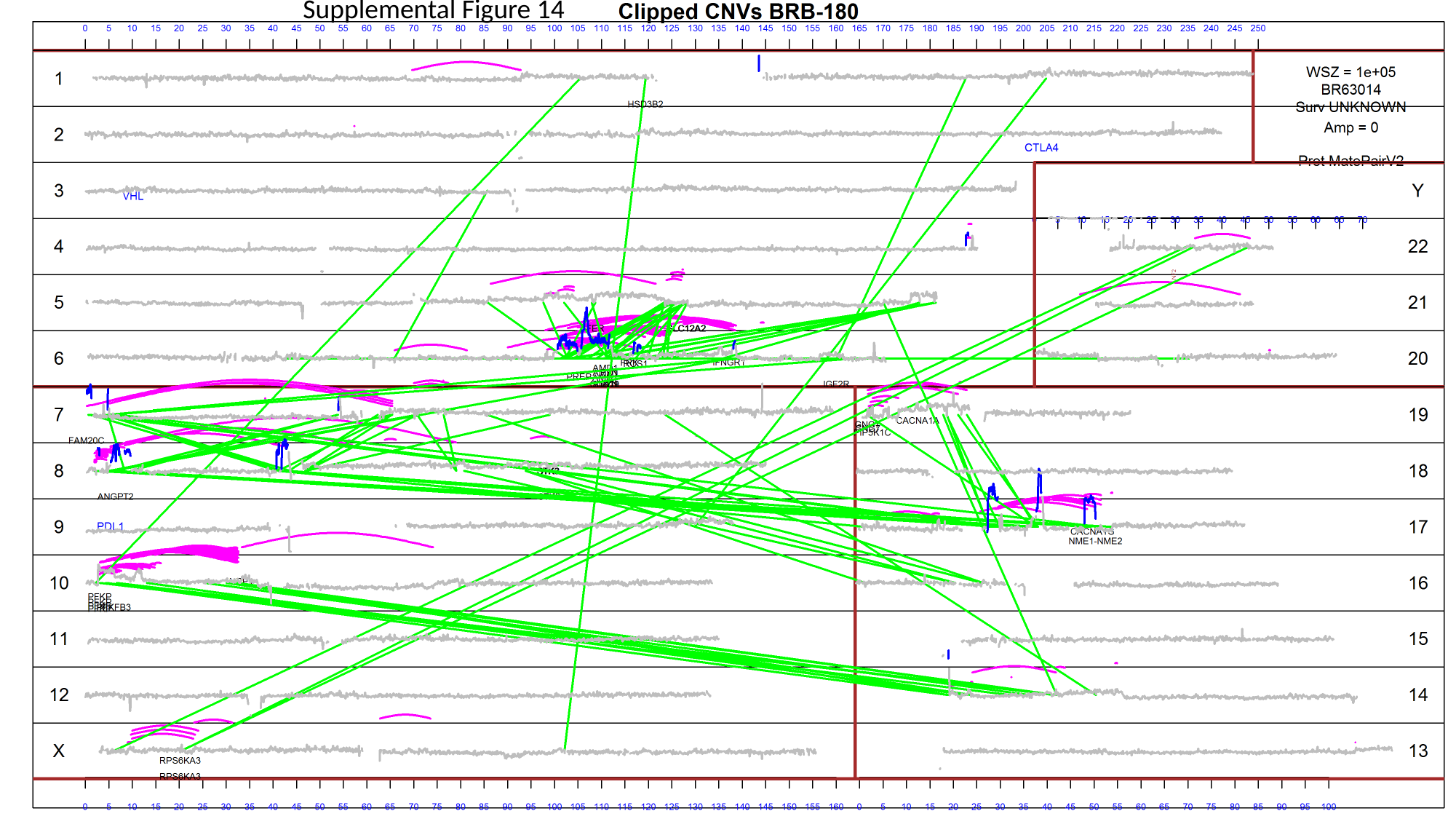

Supplemental Figure 14

## Slide 15
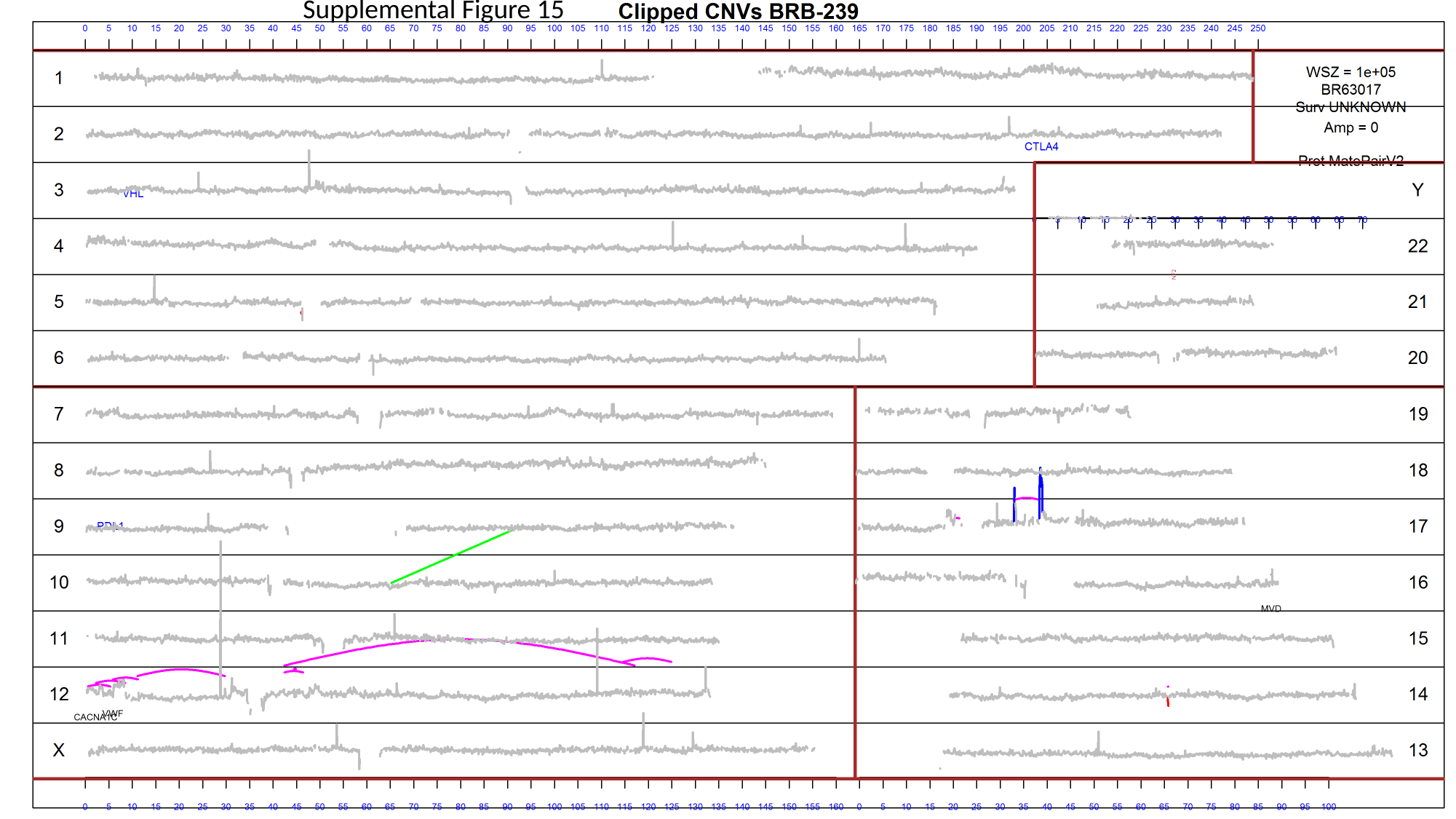

Supplemental Figure 15

## Slide 16
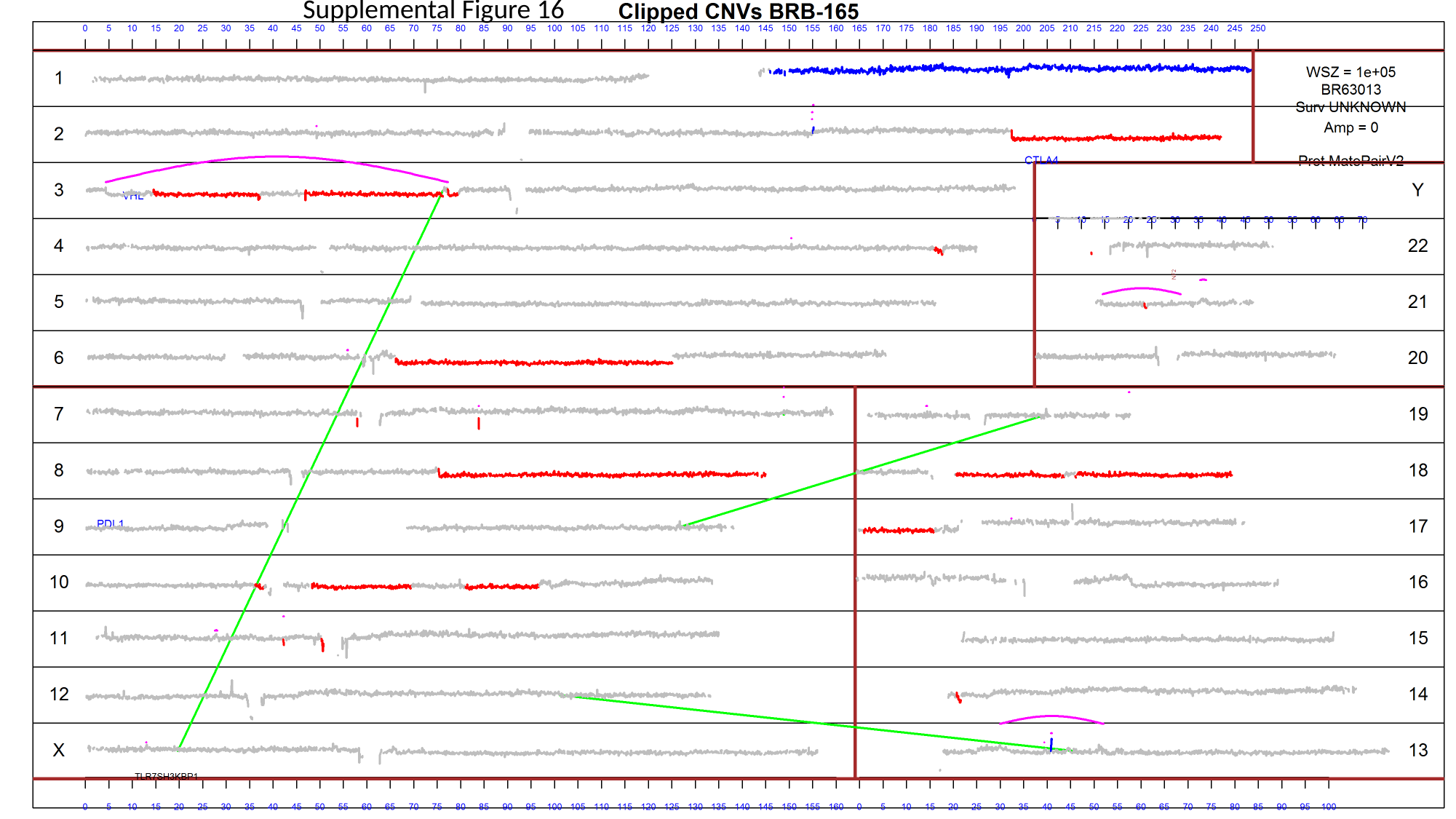

Supplemental Figure 16

## Slide 17
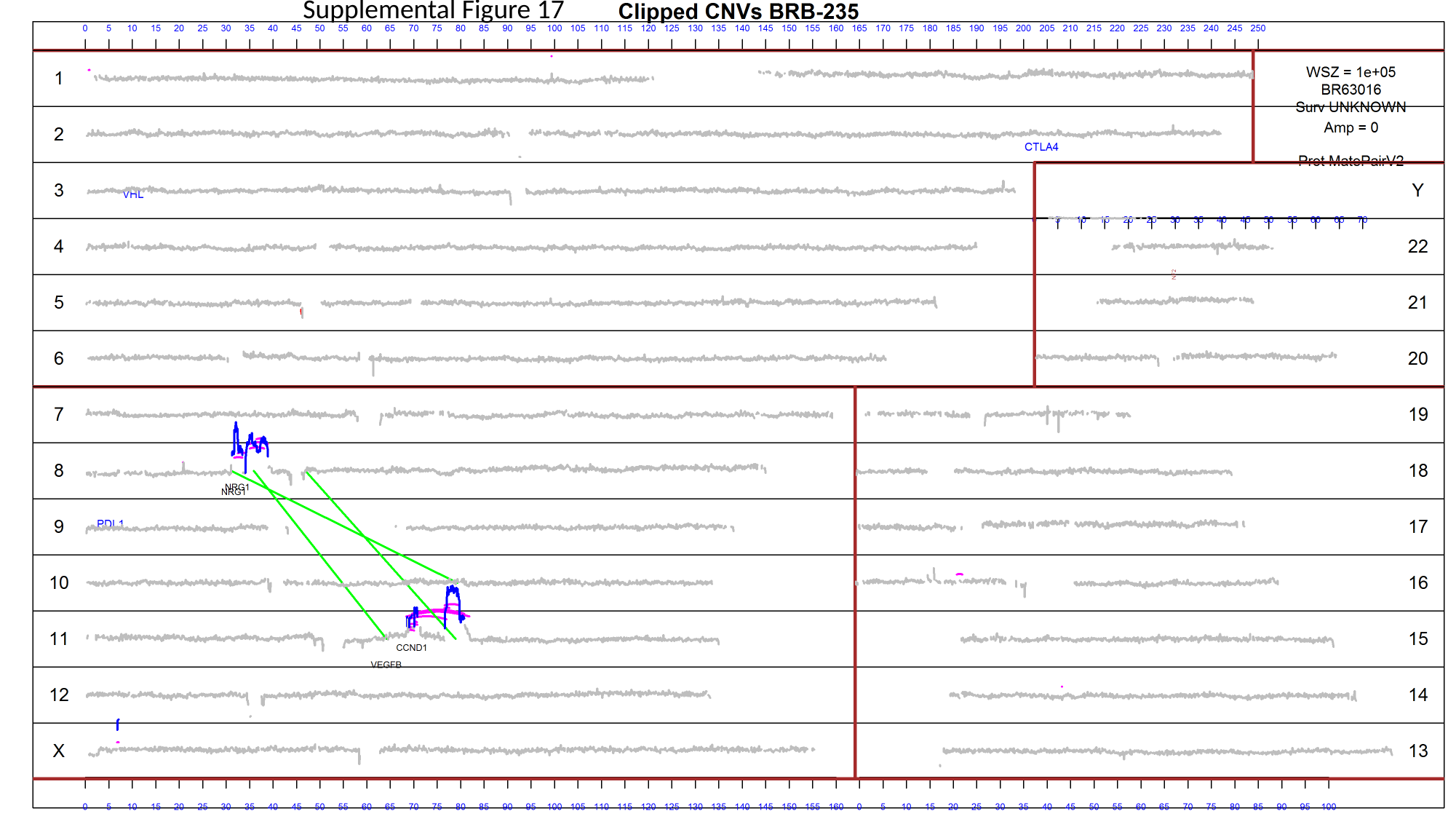

Supplemental Figure 17

## Slide 18
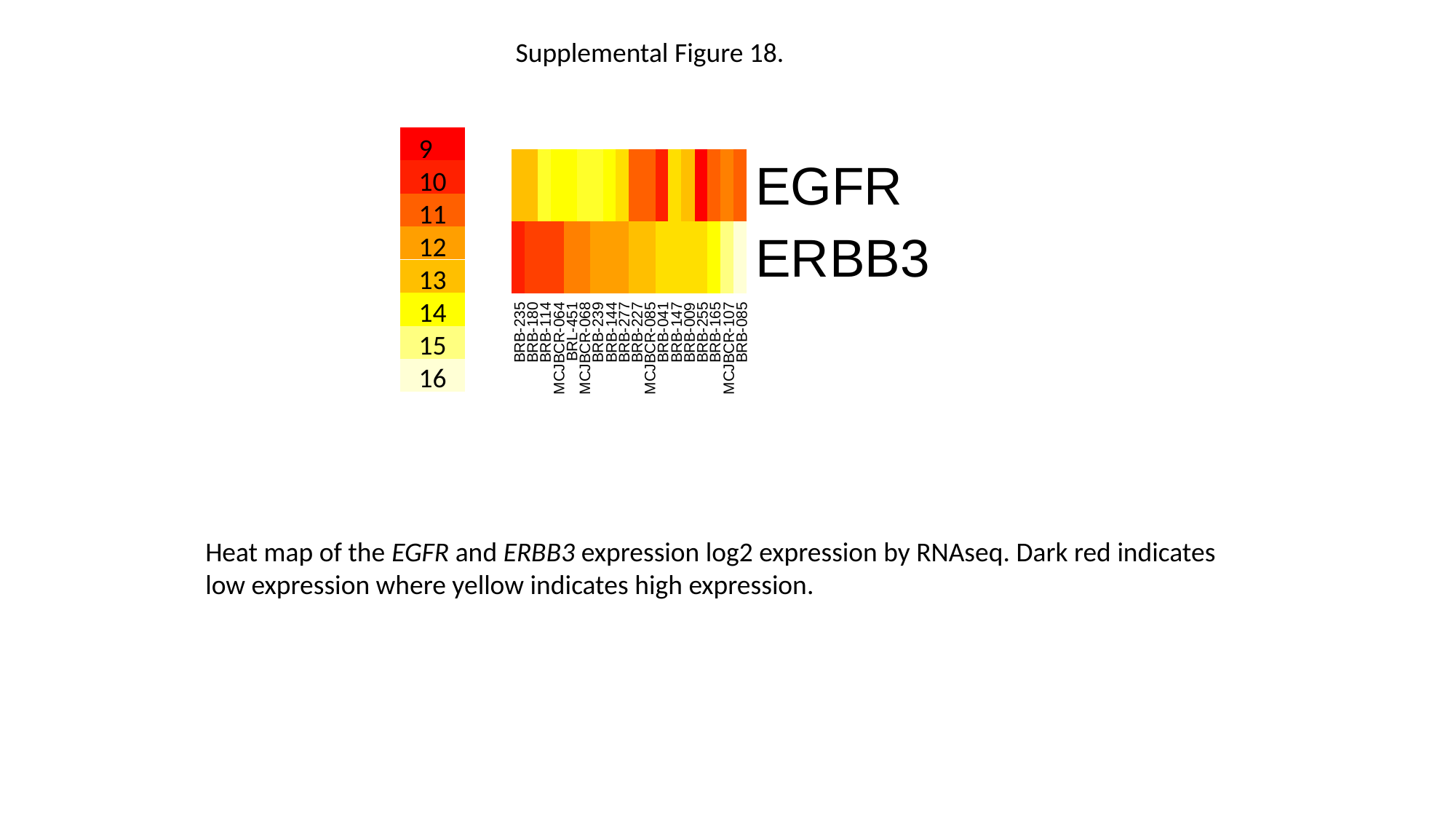

Supplemental Figure 18.
9
10
11
12
13
14
15
16
Heat map of the EGFR and ERBB3 expression log2 expression by RNAseq. Dark red indicates low expression where yellow indicates high expression.

## Slide 19
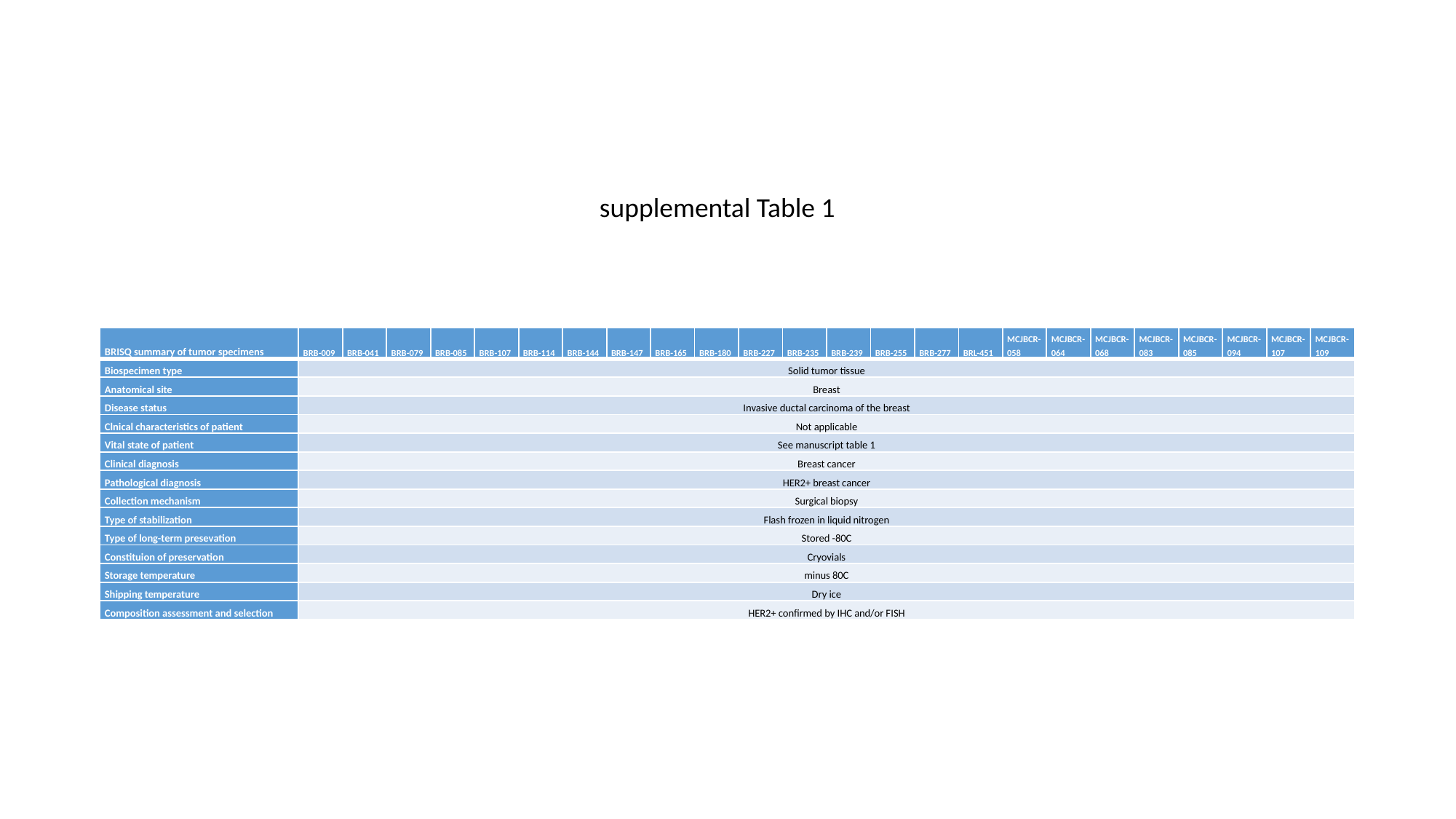

supplemental Table 1
| BRISQ summary of tumor specimens | BRB-009 | BRB-041 | BRB-079 | BRB-085 | BRB-107 | BRB-114 | BRB-144 | BRB-147 | BRB-165 | BRB-180 | BRB-227 | BRB-235 | BRB-239 | BRB-255 | BRB-277 | BRL-451 | MCJBCR-058 | MCJBCR-064 | MCJBCR-068 | MCJBCR-083 | MCJBCR-085 | MCJBCR-094 | MCJBCR-107 | MCJBCR-109 |
| --- | --- | --- | --- | --- | --- | --- | --- | --- | --- | --- | --- | --- | --- | --- | --- | --- | --- | --- | --- | --- | --- | --- | --- | --- |
| Biospecimen type | Solid tumor tissue | | | | | | | | | | | | | | | | | | | | | | | |
| Anatomical site | Breast | | | | | | | | | | | | | | | | | | | | | | | |
| Disease status | Invasive ductal carcinoma of the breast | | | | | | | | | | | | | | | | | | | | | | | |
| Clnical characteristics of patient | Not applicable | | | | | | | | | | | | | | | | | | | | | | | |
| Vital state of patient | See manuscript table 1 | | | | | | | | | | | | | | | | | | | | | | | |
| Clinical diagnosis | Breast cancer | | | | | | | | | | | | | | | | | | | | | | | |
| Pathological diagnosis | HER2+ breast cancer | | | | | | | | | | | | | | | | | | | | | | | |
| Collection mechanism | Surgical biopsy | | | | | | | | | | | | | | | | | | | | | | | |
| Type of stabilization | Flash frozen in liquid nitrogen | | | | | | | | | | | | | | | | | | | | | | | |
| Type of long-term presevation | Stored -80C | | | | | | | | | | | | | | | | | | | | | | | |
| Constituion of preservation | Cryovials | | | | | | | | | | | | | | | | | | | | | | | |
| Storage temperature | minus 80C | | | | | | | | | | | | | | | | | | | | | | | |
| Shipping temperature | Dry ice | | | | | | | | | | | | | | | | | | | | | | | |
| Composition assessment and selection | HER2+ confirmed by IHC and/or FISH | | | | | | | | | | | | | | | | | | | | | | | |
